# RISING STARS: Androgens and immune cell function

**DOI:** 10.1530/JOE-23-0398

**Published:** 2024-04-29

**Authors:** Rebecca J Ainslie, Ioannis Simitsidellis, Phoebe M Kirkwood, Douglas A Gibson

**Affiliations:** 1 Institute for Regeneration and Repair, the University of Edinburgh, Edinburgh BioQuarter, Edinburgh, United Kingdom

**Keywords:** androgen, androgen receptor, testosterone, immune system, inflammatory diseases

## Abstract

Androgens can modulate immune cell function and may contribute to differences in the prevalence and severity of common inflammatory conditions. Although most immune cells are androgen targets, our understanding of how changes in androgen bioavailability can affect immune responses is incomplete. Androgens alter immune cell composition, phenotype, and activation by modulating the expression and secretion of inflammatory mediators or by altering the development and maturation of immune cell precursors. Androgens are generally associated with having suppressive effects on the immune system, but their impacts are cell and tissue context-dependent and can be highly nuanced even within immune cell subsets. In response to androgens, innate immune cells such as neutrophils, monocytes, and macrophages increase the production of the anti-inflammatory cytokine IL-10 and decrease nitric oxide production. Androgens promote the differentiation of T cell subsets and reduce the production of inflammatory mediators, such as IFNG, IL-4 and IL-5. Additionally, androgens/androgen receptor can promote the maturation of B cells. Thus, androgens can be considered as immunomodulatory agents, but further work is required to understand the precise molecular pathways that are regulated at the intersection between endocrine and inflammatory signals. This narrative review focusses on summarising our current understanding of how androgens can alter immune cell function and how this might affect inflammatory responses in health and disease.

## Invited Author’s profile


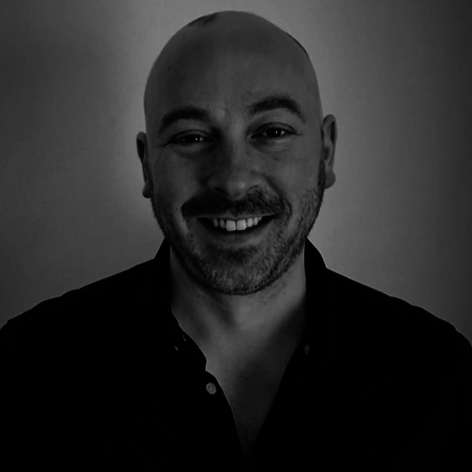


**Dr Douglas Gibson** is a principal investigator and Sir Henry Dale Fellow in the Institute for Regeneration and Repair at the University of Edinburgh. His research focusses on understanding how hormones, particularly androgens, control immune cell function in the womb and how this might affect women’s reproductive health. His lab uses fate-mapping techniques, transcriptomics analysis, multiparameter flow cytometry and immunohistochemistry to characterise the phenotype and function of immune cell populations that are required for endometrial repair and remodelling.

## Introduction

Androgens play important roles in human health, including the development and maintenance of the reproductive system, regulating cardiovascular function and metabolism (Davey & Grossmann 2016). Androgen signalling has broad physiological effects, but how it regulates the immune system is poorly understood.

Evidence for the impact of androgens on immune cells comes from *in vitro* assessment of isolated populations or deduced from cohort differences due to sex or suppression of endogenous hormones. It is well known that sexual dimorphism in immune responses affects the prevalence and severity of a range of inflammatory conditions, but our understanding of the specific contribution of androgens to immune responses and the impact androgens may have on different immune cell subsets is incomplete. Interpretation is limited by the attribution of male-predominant responses to androgens and female-predominant responses to estrogens. Moreover, the potential for androgens to be aromatised to estrogens is often not controlled for in experimental studies.

Immune cell populations from both the innate and adaptive immune systems express the androgen receptor (AR) and may therefore be sensitive to fluctuations in androgen bioavailability, which may change with age, sex and as a result of endocrine disorders. This narrative review focusses on how androgens regulate immune cell phenotype and function, and how this affects inflammatory responses in health and disease.

### Search method

A review of published literature was performed using PubMed to search for articles and reviews containing the following main keywords: androgens, AR, testosterone, immune system, immune cells, sexual dimorphism, and other key terms related to these subjects.

## Androgens and androgen signalling

Androgens are sex steroid hormones that are classically considered ‘male’ due to their important roles in the development and maintenance of the male reproductive system (Barsoum & Yao 2006). However, androgens also play important roles in regulating the function of various tissues and organs in both sexes, including muscle, heart, kidney and bone (Davey & Grossmann 2016), as well as the female reproductive tract (Simitsidellis *et al.* 2018). They are predominantly produced by the gonads (testes and ovaries) and the adrenal glands (Bienenfeld *et al.* 2019) but are also produced through the conversion of precursors in peripheral tissue sites, such as adipose tissue. The bioavailability of androgens can vary with age and different developmental and endocrine disorders, resulting in high (hyperandrogenism) or low (androgen deficiency) systemic androgen concentrations (Jordan & DonCarlos 2008). Androgen-related disorders can be caused by different mechanisms, including defects in androgen synthesis, metabolism or receptor signalling (Khera & Lipshultz 2006).

Androgens consist of both inactive precursors, dehydroepiandrosterone sulphate (DHEAS), dehydroepiandrosterone (DHEA) and androstenedione (A4), and active androgens, namely, testosterone and dihydrotestosterone (DHT). In addition, recent evidence supports an emerging role for the importance of 11-oxygenated androgens, a group of androgen metabolites that are present in the circulation at concentrations that can be equivalent to those of ‘classical’ androgens and can bind the androgen receptor in target tissues (Alemany 2022, Storbeck & O’Reilly 2023).

The *de novo* synthesis of androgens requires sequential enzymatic modification of cholesterol to generate active androgens. This is mediated by cytochrome P450 enzymes, hydroxysteroid dehydrogenases and aldo/keto reductases (Manenda *et al.* 2016), and their differential expression throughout the body regulates local androgen action. Testosterone is the main circulating androgen in men, and it can be converted within target tissues to the potent androgen DHT by 5α-reductase or aromatised into 17β- estradiol by aromatase, therefore contributing to both androgenic and estrogenic effects in tissues.

Upon reaching the target tissue, testosterone can passively diffuse across the cell membrane or exert its actions at the cell surface (Oren *et al.* 2004). Additionally, androgens can act in an intracrine manner where activation and action occur within target tissues via activation of inactive precursors such as DHEAS, DHEA and A4. Intracrine androgen action has been documented in a range of tissues, including the prostate, adipose tissue and the endometrium (Schiffer *et al.* 2018, Simitsidellis *et al.* 2018). Notably, intracrine androgen action has been proposed to affect immune cell function and has been described in human alveolar macrophages (Milewich *et al.* 1983) and synovial macrophages (Cutolo *et al.* 1996), as well as murine T lymphocytes (Samy *et al.* 2001).

### Mechanisms of androgen signalling

Classically, androgen signalling is mediated through specific binding and activation of the androgen receptor (AR) protein (reviewed in Davey & Grossmann (2016)). AR is primarily located in the cytoplasm where, in the absence of androgens, it is found in a complex with heat shock and chaperone proteins, such as HSP70 (Bennett *et al.* 2010).

### Direct AR signalling

**Figure 1:**
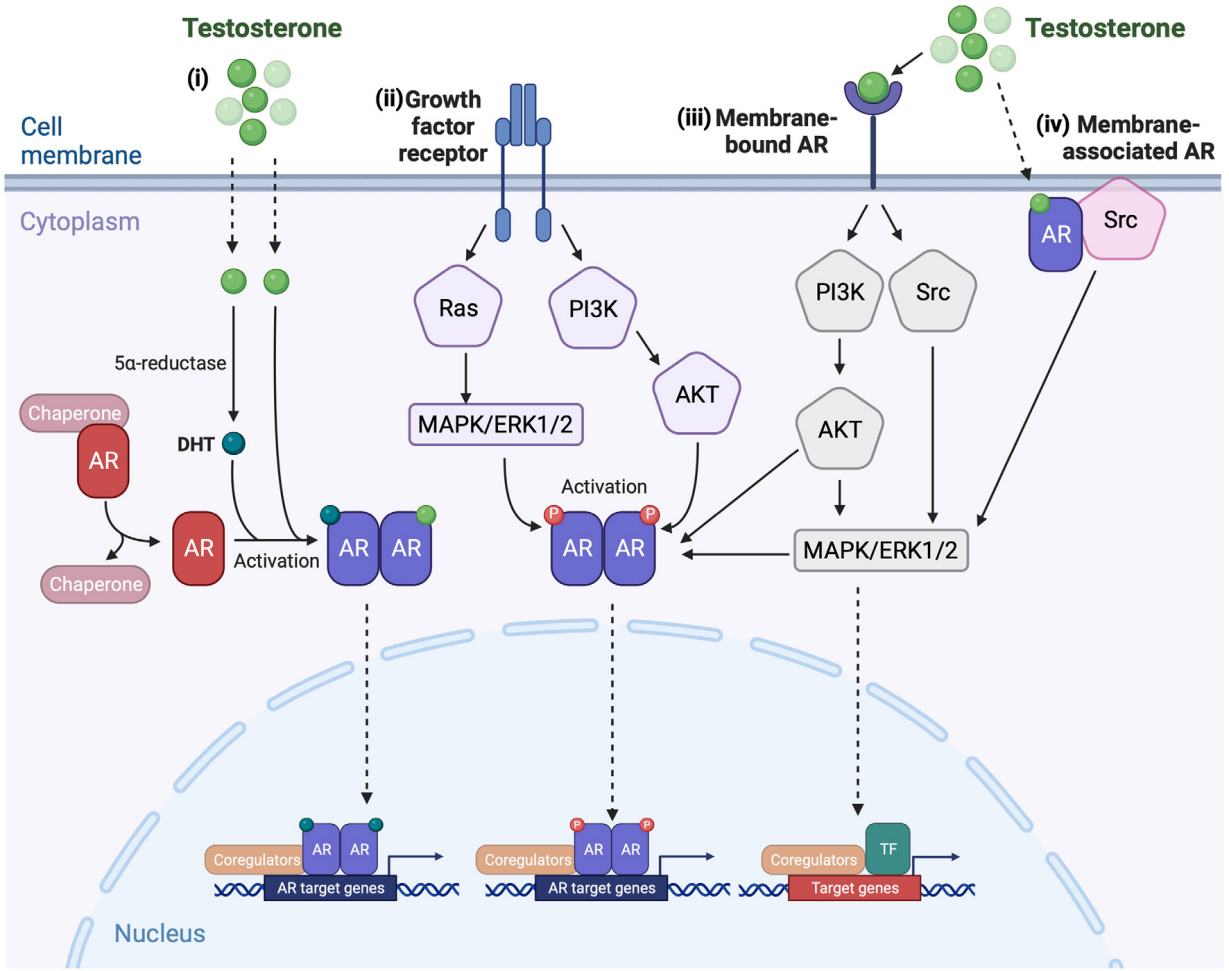
Direct and indirect mechanisms of androgen signalling. (i) Ligand-dependent direct androgen receptor signalling: Upon androgen binding to the cytoplasmic androgen receptor (AR), AR begins to dissociate from the chaperone proteins and translocates to the nucleus. Once inside the nucleus AR binds to androgen target genes through androgen response elements to alter the transcription of target genes (Davey & Grossmann 2016). (ii) Ligand-independent androgen receptor signalling: Cytoplasmic AR can be activated through phosphorylation due to activation of growth factor receptor mediated signalling pathways. The phosphorylated AR is then able to translocate to the nucleus and induce the expression of androgen target genes (Bennett *et al.* 2010, Davey & Grossmann 2016, Kadel & Kovats 2018). (iii) Indirect membrane-bound androgen receptor signalling: Androgens can bind to a membrane bound form of the AR and induce signalling transduction which results in the binding of transcription factors to target genes altering gene expression. This induction of intracellular signalling pathways can also result in the phosphorylation of the cytoplasmic form of the AR which can then translocate to the nucleus and induce AR-dependent direct signalling pathway (Kousteni *et al.* 2001, Estrada *et al.* 2003, Kang *et al.* 2004, Davey & Grossmann 2016, Thomas 2019). (iv) Indirect membrane associated androgen receptor signalling: Androgens can also exert their effects via the cytoplasmic AR that is localised at the cell membrane. Binding of androgens to this receptor leads to activation of intracellular signalling pathways that result in the transcription of target genes (Kousteni *et al.* 2001, Estrada *et al.* 2003, Kang *et al.* 2004, Davey & Grossmann 2016, Thomas 2019). AKT, protein kinase B; AR, androgen receptor; DHT, dihydrotestosterone; ERK1/2, extracellular signal-regulated kinase 1/2; MAPK, mitogen-activated protein kinases; PI3K, phosphoinositide 3-kinases; Ras, rat sarcoma; Src, proto-oncogene tyrosine-protein kinase; TF, transcription factor. Created with BioRender.com.

Following androgen binding, the androgen/AR complex alters its conformation, dissociates from chaperone proteins, and translocates to the nucleus where it binds as a dimer to consensus androgen response element regions in DNA. Transcriptional regulation of target gene expression is modulated by co-regulatory proteins that form complexes with the activated AR to enhance or repress transcription (Fig. 1(i), (Davey & Grossmann 2016)). Specifically, regulation of gene expression by AR can be mediated via interaction with general transcription factors (such as SRC-1 and TF_II_H), components of the chromatin remodelling complex (such as SWI/SNF), histone-modifying enzymes and proteins involved in RNA splicing and metabolism (for a comprehensive review on the subject see Heemers & Tindall (2007)). AR-dependent signalling can also occur independently of androgens via activation of growth factor receptors and downstream signalling pathways that promote direct phosphorylation and activation of cytoplasmic AR (Fig. 1(ii)) (Davey & Grossmann 2016). This activation promotes nuclear translocation of the AR and transcriptional regulation (Kadel & Kovats 2018). This mechanism of ligand-independent AR signalling has been described in prostate cancer where upregulation of androgen target genes occurs through pathways such as the protein kinase A (PKA), proteins kinase C and mitogen-activated protein kinases (MAPK) signalling pathway in the presence of non-androgen ligands, such as IL-6 (Lyons *et al.* 2008, Davey & Grossmann 2016).

### Indirect AR signalling

Several studies have identified a further mechanism of androgen action that occurs too rapidly (seconds to minutes rather than hours to days) to be via a direct mechanism (Davey & Grossmann 2016). This led to the discovery of membrane-bound AR that can be found in a range of cell types including Sertoli cells (Gorczynska & Handelsman 1995), prostate cancer cells (Hatzoglou *et al.* 2005, Sun *et al.* 2006), satellite cells from human skeletal muscles (Sinha-Hikim *et al.* 2004) and some immune cell types such as T lymphocytes (Benten *et al.* 1999 *a*) and macrophages (Benten *et al.* 1999*b*). Indirect androgen signalling can occur through direct binding of androgens to membrane-bound ARs or via cytoplasmic ARs that are tethered to the cell membrane (Fig. 1(iii) and (iv)), (Thomas 2019)). The binding of androgens to these receptors leads to the release of intracellular second messengers such as calcium and/or activation of a range of signalling pathways including extracellular signal-regulated kinase (ERK), protein kinase B (PKB or Akt) and MAPK (Migliaccio *et al.* 2000, Kousteni *et al.* 2001, Estrada *et al.* 2003, Kang *et al.* 2004). The activation of these signalling pathways results in androgen-dependent changes in the expression of target genes without direct binding of the AR to DNA (Thomas 2019).

Due to variations in ligand bioavailability, as well as different cell- and tissue-specific expressions of coregulatory proteins, the impact of androgens must be investigated in a context-specific way. Thus, the different cell types, AR expression/localisation and concomitant inflammatory signals must all be accounted for when seeking to understand how androgens affect immune cell function.

## The immune system

The immune system is a complex network of cells, proteins and organs that function to protect the body from infection and disease, as well as aid in the repair of damaged tissues. The immune system is broadly divided into two parts: the innate immune system and the adaptive immune system (Marshall *et al.* 2018).

### Innate immune system

The innate immune system is the body’s first line of defence against invading pathogens and is therefore highly responsive and fast-acting (within minutes of exposure). The innate immune system is comprised of a range of immune cells including monocytes, macrophages, dendritic cells (DCs), mast cells, neutrophils, eosinophils, basophils, natural killer cells and innate lymphoid cells (Turvey & Broide 2010). During infection, resident phagocytic cells, such as macrophages and DCs, identify pathogens through the recognition of foreign cell-surface markers, which leads to encapsulation of the pathogen through a process known as phagocytosis. This internalisation event stimulates the release of a cascade of pro-inflammatory mediators known as cytokines and chemokines. These proteins initiate a state of inflammation in the local area which attracts other immune cells, such as neutrophils and monocytes, that aid in the clearance of an infection through direct killing and orchestration of the immune response. Another important component of innate immunity is the propagation of a prolonged immune response through the activation of the adaptive immune system by antigen-presenting cells (APCs), such as macrophages and DCs (Murphy & Weaver 2016).

### Adaptive immune system

The adaptive immune system is a secondary line of defence that is initiated by cells of the innate immune system when an innate immune response fails to clear a new infection. The adaptive immune system is composed of two main cell types (T lymphocytes and B lymphocytes) that have the ability to mount an antigen-specific response against pathogens (Murphy & Weaver 2016). Adaptive immunity also has the capacity to remember exposure to previous pathogens, enabling a rapid and more efficient response upon future exposure to the same or similar pathogens. T lymphocytes have important roles in the direct killing of infected cells, activation of other immune cells and regulation of the immune response. B lymphocytes produce antibodies against foreign antigens, and unlike T lymphocytes, they can recognise antigens directly through unique antibodies expressed on their surface without the assistance of APCs (Marshall *et al.* 2018).

### Sexual dimorphism in immunity

It is well established that sexual dimorphism (differences between males and females) exists within the immune system (Shepherd *et al.* 2021), which impacts responses to infection as well as the risk of autoimmune diseases (Cook 2008, McCombe *et al.* 2009, Zuk 2009). In general, women can generate greater innate and adaptive immune responses than men (Klein & Flanagan 2016). This is associated with reduced rates of bacterial, viral and parasite infections in women and a higher rate of survival for several cancer types compared to men (Jung *et al.* 2012). In contrast, there is a greater prevalence of autoimmune disease in women; approximately 80% of all patients with autoimmune diseases are women (Angum *et al.* 2020). This is consistent with heightened immune reactogenicity towards molecular patterns that originate from both self and non-self in females (Shepherd *et al.* 2021). The key drivers of sexual dimorphism are genetics (chromosomal sex) and sex hormones. We know that autoimmune diseases have a complex genetic basis and that associations between genetics and autoimmune diseases include the presence of certain major histocompatibility complex genes (Taneja 2021) and the expression of toll-like receptors (TLRs), which are encoded by the X chromosome (Rubtsova *et al.* 2015). This review focusses on the association between androgens and the immune system; details of the effects of genetics and other sex hormones (estrogens) on sexual dimorphism in the immune system are reviewed elsewhere (Rubtsova *et al.* 2015, Moulton 2018, Taneja 2021, Harding & Heaton 2022, Sciarra *et al.* 2023).

### Androgen receptor expression in the immune system

**Figure 2:**
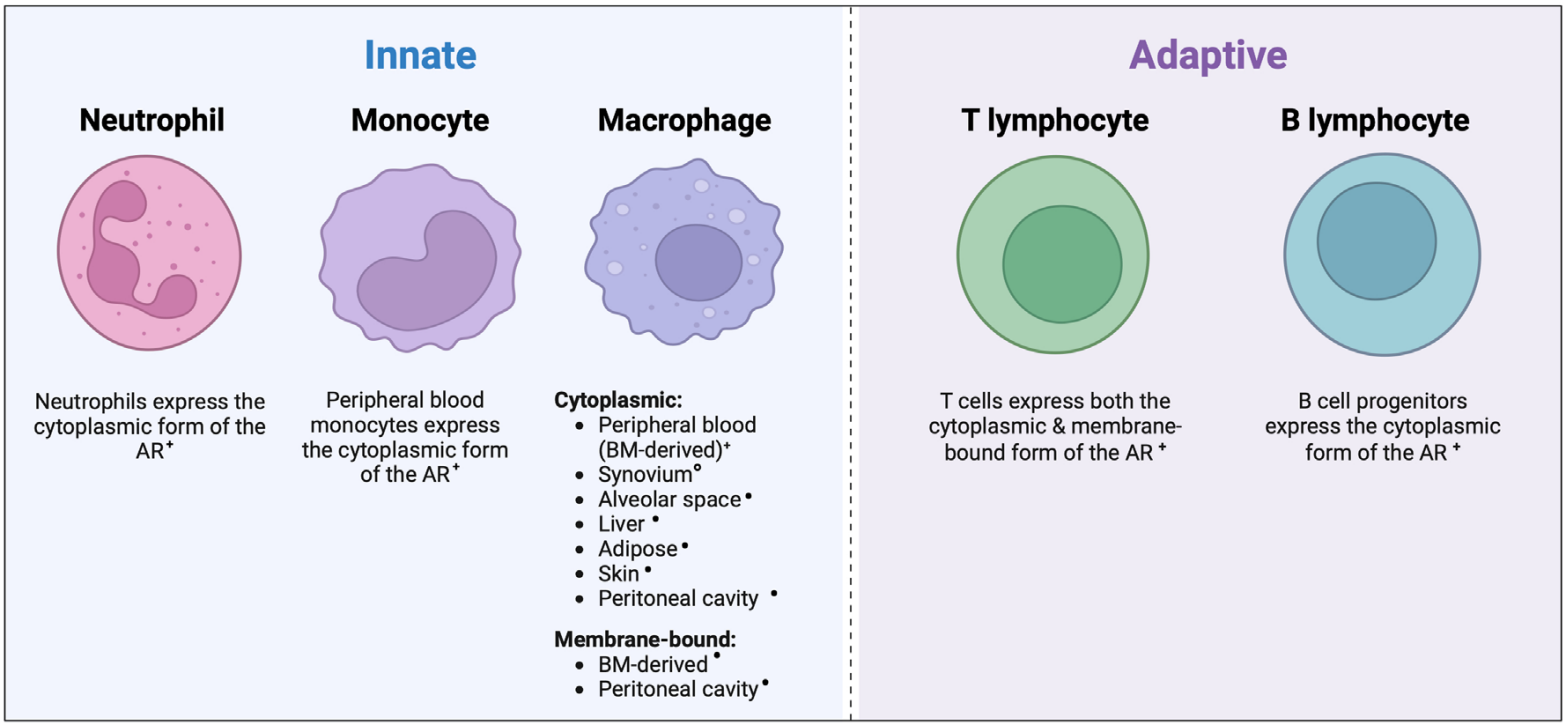
Androgen receptor expression in immune cells. Innate immune cells such as neutrophils, monocytes and macrophages express AR. AR is predominantly expressed in the cytoplasm with some evidence for membrane expression in macrophage subsets. T and B lymphocytes of the adaptive immune system are also AR positive with AR detected in the cytoplasm and membrane of T cells and cytoplasm of B cell progenitors (Milewich *et al.* 1983, Kovacs & Olsen 1987, Viselli *et al.* 1997, Benten *et al.* 1999*a*, Mantalaris *et al.* 2001, Wunderlich *et al.* 2002, Liu *et al.* 2004, Sinha-Hikim *et al.* 2004, Liu *et al.* 2005, Ahmadi & McCruden 2006, Bolego *et al.* 2013, Bizzaro *et al.* 2018, Rubinow *et al.* 2018, Lee *et al.* 2019, Markman *et al.* 2020, McCrohon *et al.* 2000, Campesi *et al.* 2012). •, mouse; °, human; +, mouse and human. Created with BioRender.com.

Immune cells from both the innate and adaptive immune system express AR (summarised in Fig. 2), where receptor protein expression is commonly localised to the cytoplasm and, in some cases, the cell membrane. Flow cytometry, immunofluorescence and gene expression analyses have been predominantly used to determine AR expression in macrophage subsets of humans and mice (Cutolo *et al.* 1996, Benten *et al.* 1999 *b*, Mantalaris *et al.* 2001, Wunderlich *et al.* 2002, Liu *et al.* 2004, 2005, Ahmadi & McCruden 2006, Bizzaro *et al.* 2018, Rubinow *et al.* 2018, Lee *et al.* 2019, McCrohon *et al.* 2000, Campesi *et al.* 2012). These studies revealed the presence of either cytoplasmic or membrane-bound AR in subsets of murine macrophages and confirmed the expression of cytoplasmic AR in bone marrow-derived and synovial macrophages in humans (Cutolo *et al.* 1996, Benten *et al.* 1999*b*, Mantalaris *et al.* 2001, Wunderlich *et al.* 2002, Liu *et al.* 2005). RT-PCR analysis identified *AR* mRNA expression in human peripheral blood monocytes and the monocyte THP-1 cell line (Liu *et al.* 2004). *AR* expression has not been comprehensively described in neutrophils, but cytoplasmic AR protein has been detected by immunohistochemistry throughout stages of neutrophil development in humans (Mantalaris *et al.* 2001, Markman *et al.* 2020).

Investigations into expression of AR in the adaptive immune system have involved the use of binding assays, flow cytometry and western blot analysis. Benten *et al.* assessed the binding of the conjugated ligand testosterone–BSA–FITC, which cannot cross the cell membrane, combined with confocal microscopy and flow cytometry to demonstrate ligand binding to membrane AR in murine CD4^+^ and CD8^+^ T cells (Benten *et al.* 1999*a*). Further *in vitro* studies also confirmed the expression of cytoplasmic AR in human and murine T cells (Kovacs & Olsen 1987, Benten *et al.* 1999*a*). A combination of techniques, including western blot, RT-PCR, flow cytometry and confocal microscopy, have been used to investigate the expression of AR in murine B cells and their progenitors, leading to the identification of cytoplasmic AR expression in B cell progenitors. Notably, AR was absent in peripheral or mature B cells (Viselli *et al.* 1997, Benten *et al.* 2002*b*) consistent with a potential role for AR in B cell development.

In addition to the expression of AR in mature immune cells, haematopoietic stem cells, common myeloid progenitors (CMPs) and common lymphoid progenitors (CLPs) in bone marrow also express AR (Mierzejewska *et al.* 2015). Due to the expression of AR across these cell types, androgen signalling may have an important role in regulating the immune system, affecting both immune cell development and/or the function of mature immune cells.

### Impact of androgens on the innate immune system

#### Monocytes and macrophages

Monocytes and macrophages are innate immune cells with important roles in the initiation and maintenance of a non-specific immune response, as well as the activation of the adaptive immune system (Murphy & Weaver 2016). Monocytes and macrophages are mononuclear phagocytes that play distinct roles in immunity, with monocytes having important roles in orchestrating inflammation as well as protecting the body from pathogens, while tissue-resident macrophages are key players in tissue homeostasis and the resolution of inflammation. Monocytes are continuously replenished from the bone marrow, where they are derived from haematopoietic stem cells. These cells are released from the bone marrow and travel around the body to target tissues where they can then differentiate into macrophages (Ginhoux & Jung 2014). As seen in Fig. 2, peripheral blood monocytes in both humans and mice express the cytoplasmic form of the AR. AR expression and localisation (cytoplasmic or membrane) in macrophages varies depending on tissue context in both humans and mice.

Several animal studies have investigated the impact of androgens on monocyte and macrophage function. These studies, summarised in Table 1, have utilised *in vitro* experiments where different macrophage cell lines are exposed to exogenous androgens in the presence or absence of lipopolysaccharide (LPS) stimulation to investigate the impact of androgens on macrophage activation. One study compared unstimulated macrophages with LPS-stimulated macrophages from the murine cell line J774 to investigate the effects of testosterone on macrophage cytokine production. Testosterone treatment induced the production of interleukin 10 (IL-10) in unstimulated macrophages, and this production was significantly increased in LPS-stimulated macrophages. Treatment with testosterone in stimulated macrophages also led to reduced production of nitric oxide (NO) and tumour necrosis factor alpha (TNF-α) (D’Agostino *et al.* 1999). Similarly, when the macrophage cell line RAW 264.7 was exposed to increasing concentrations of testosterone (0.1–40 µM) and stimulated with LPS, testosterone had a dose-dependent inhibitory effect on the protein expression of NO and both the protein and mRNA expression of NO synthase (NOS) (Friedl *et al.* 2000). Further studies in which peritoneal macrophages were cultured with testosterone or DHT identified that androgen treatment led to increased gene expression of IL-6 (*IL6*) but had no effect on the expression of *TNF* and transforming growth factor beta 1 (*TGFB1)* (Gilliver *et al.* 2006). Furthermore, testosterone-induced apoptosis of bone marrow-derived macrophages increased both the mRNA and protein expression of caspase 3, caspase 8 and poly (ADP-ribose) polymerase (PARP) as determined by RT-PCR and western blot (Jin *et al.* 2006). Treatment of RAW 264.7 cells with testosterone led to a decrease in TLR4 protein expression consistent with androgens priming a reduced response to inflammatory stimuli that act through TLR4 (Rettew *et al.* 2008).

**Table 1:** A summary of primary research studies investigating the effects of androgens on murine macrophages *in vitro*.

Species	Cell type	Source	Sex	Purpose	Experimental design	*In vitro*	Key findings	Analysis technique	Reference
Mouse	Macrophage J774 cell line	Ascites	Female	Investigate the effects of testosterone on murine macrophage production of cytokine	Murine macrophages were cultured with testosterone for 24–48 h and cytokine production was measured	*In vitro*	Testosterone treatment induced spontaneous or LPS-mediated production of IL-10 and reduction of NO and TNF	ELISA and Griess reaction	D’Agostino *et al.* (1999)
Mouse	Macrophage RAW 264.7 cell line	Cancer (Abelson leukaemia virus)	Male	Investigate the effect of testosterone on inducible NO synthesis in murine macrophages	Murine macrophages were stimulated with LPS and were exposed to increasing levels of testosterone (0.1–40 µM)	*In vitro*	Testosterone treatment led to a dose- dependent inhibition of NO and NOS	Griess reaction and Immunoblotting	Friedl *et al.* (2000)
Mouse	Macrophage	Peritoneal cavity	Male	To investigate the effect of testosterone or DHT on murine macrophages	Peritoneal macrophages were cultured with testosterone or DHT and cytokine production was measured	*In vitro*	Administration of testosterone and DHT led to an increase in the expression of the *IL6* gene but had no effect on TNF and TGFB1	ELISA	Gilliver *et al.* (2006)
ND	Macrophage	Bone marrow	ND	To investigate the effect of testosterone on the apoptosis of BM-derived macrophages	BM-derived macrophages were stimulated with testosterone in the presence or absence of M-CSF	*In vitro*	Administration of testosterone can induce apoptosis of BM-derived macrophages, increased expression of caspase 3, caspase 8 and PARP. Blockade of FADD (upstream of caspase 8 in the FAS/FASL pathway) led to reduced expression of caspase 8 and apoptosis	Flow cytometry, RT-PCR and western blot	Jin *et al.* (2006)
Mouse	Macrophage RAW 264.7 cell line	Cancer (Abelson leukaemia virus)	Male	Investigate the effect of testosterone on the expression of TLR4 on murine macrophages	Murine macrophages were exposure to various levels of testosterone propionate (1–1000 nM) for different time periods	*In vitro*	Administration of testosterone led to a decrease in TLR4 expression. These results were corroborated in peritoneal macrophages from orchiectomised mice	Flow cytometry	Rettew *et al.* (2008)

BM, bone marrow; DHT, dihydrotestosterone; ELISA, enzyme-linked immunosorbent assay; FADD, FAS-associated death domain; FAS, FAS cell surface death receptor; FASL, Fas ligand; IL-6, interleukin 6; IL-10, interleukin 10; LPS, lipopolysaccharide; M-CSF, macrophage colony-stimulating factor; ND, not described; NO, nitric oxide; NOS, nitric oxide synthase; PARP, poly (ADP-ribose) polymerase; RT-PCR, reverse transcription polymerase chain reaction; TGFB1, transforming growth factor beta 1; TLR4, toll-like receptor 4; TNF, tumour necrosis factor.

In summary, these studies (Table 1) demonstrate that androgens, in general, promote an anti-inflammatory macrophage phenotype through the upregulation of key anti-inflammatory mediators, such as IL-10, reduced production of pro-inflammatory mediators, such as NO and TNF, and decreased expression of TLRs (D’Agostino *et al.* 1999, Friedl *et al.* 2000, Gilliver *et al.* 2006, Jin *et al.* 2006, Rettew *et al.* 2008).

#### Dendritic cells

DCs are an innate immune cell type that bridges the gap between innate and adaptive immune responses. The main function of DCs is to digest phagocytosed pathogens to process and present foreign antigens to adaptive immune cells (Martin-Gayo & Yu 2019). For the most part, studies investigating the expression of AR on DCs are either lacking or have utilised western blot analysis to demonstrate that myeloid-derived DCs do not express AR (Paharkova-Vatchkova *et al.* 2004). However, more recent chromatin immunoprecipitation sequencing analysis of tumour-associated DCs as well as splenic DCs identified AR expression in these cell types (Thompson *et al.* 2017). Further studies are required to definitively confirm the expression of AR on DC cell subsets.

Regardless, functional studies suggest that DC activity can be androgen-regulated. One *in vitro* study utilised murine bone marrow-derived DCs (BMDCs) and exposed them to LPS and varying concentrations of DHT (1 –500 nM) to investigate the effect of DHT on BMDC function. After 24 h, it was demonstrated that there was a dose-dependent downregulation of IL-6 as well as an upregulation in the expression of TNF, IL-10 and IL-4 in BMDCs exposed to 50–500 nM of DHT (Thompson *et al.* 2017). A study investigating DCs from hypogonadal men found that CD16+ DCs stimulated with CpG oligodeoxynucleotides had significantly higher expression levels of the activation marker CD107b compared to healthy controls consistent with androgens inhibiting DC activation (Corrales *et al.* 2012). Collectively, these limited studies suggest androgens promote an anti-inflammatory DC phenotype.

### Impact of androgens on monocytes and macrophages in disease

#### Wound healing

Macrophages play an important role in the regulation of tissue regeneration and wound healing (Martin & Leibovich 2005). Biological sex contributes to age-related differences in wound healing, which has been attributed to differences in circulating androgens; elderly males experience delayed healing of acute wounds compared to females of the same age (Guo & DiPietro 2010).

To investigate the molecular mechanisms contributing to this difference, Lai *et al.* utilised two mouse models with genetic deletion of AR. Global AR knockout (GARKO) mice were generated by breeding transgenic mice with *loxP*-flanked AR allele (*Ar^flox^*) with mice expressing the *Cre* transgene under the control of the *Actb* promoter to generate mice that lack the AR in all cell types (*Actb^ Cre^Ar^flox^*). The second model involved breeding *Ar^flox^* mice with mice expressing the *Cre* transgene under the control of the *LyzM* promoter to generate myeloid-specific AR knockout (*LyzM^Cre^Ar^flox^*) mice. It was observed that cutaneous wound healing was accelerated in both AR knockout models. The mechanism of immunosuppression by AR in this context was due to the loss of three AR-dependent mechanisms; increased monocyte recruitment, upregulation of monocyte CCR2 expression and enhanced TNF production by macrophages (Lai *et al.* 2009). Reciprocal bone marrow transplantation experiments demonstrated that the suppressive effects of androgens on wound healing can be mediated by both androgen-dependent and -independent pathways, but the effects are exclusively mediated by AR. This was further investigated through the topical application of the anti-AR compound, ASC-19, which degrades the AR protein. This treatment accelerated cutaneous wound healing and dampened TNF production (Lai *et al.* 2009). Taken together, these data illustrate that androgens have a suppressive effect on wound healing and that targeting the AR directly may present a better therapeutic option for blocking this suppression than altering systemic androgen concentrations.

#### Asthma

Asthma is a chronic heterogeneous disease of the lungs that results in restricted airflow and respiratory problems (Papi *et al.* 2020). There are sex differences in both disease prevalence and the phenotype of asthma (Laffont *et al.* 2017), such that adult women have an increased prevalence and severity of asthma compared to adult men (Chowdhury *et al.* 2021). This sex difference could be partly attributed to a protective effect of greater circulating androgen concentrations in males, which can act as bronchodilators and immunosuppressors (Montaño *et al.* 2020). In a mouse model of allergic lung inflammation, castrated male mice administered with a DHT pellet had reduced inflammation compared to castrated mice that received a placebo pellet due to reduced immune cellularity in bronchoalveolar lavage fluid (Becerra-Díaz *et al.* 2018). Alveolar macrophages have important roles in suppressing inflammation in the lung and maintaining homeostasis (Draijer & Peters-Golden 2017). Studies using the same myeloid-specific ARKO mouse model (*LyzM^Cre^AR^flox^*) described previously revealed that loss of myeloid AR in males, but not females, led to a reduction in eosinophil recruitment as well as lung inflammation due to impaired polarisation of anti-inflammatory alveolar macrophages (Becerra-Díaz *et al.* 2018). Interestingly, testosterone was investigated as an experimental asthma treatment in the 1960s which showed improved asthma symptoms in 88% of women with asthma, although the mechanisms were not elucidated (Wulfsohn *et al.* 1964). The current literature is consistent with androgens and the AR modulating myeloid cell function by inducing an anti-inflammatory phenotype and contributing to tissue repair during lung inflammation.

#### Polycystic ovarian syndrome

Polycystic ovarian syndrome (PCOS) is a complex disorder that affects 4–20% of women of reproductive age worldwide (Deswal *et al.* 2020). Seventy to eighty percent of women with PCOS have hyperandrogenism (Baptiste *et al.* 2010, Huang *et al.* 2010), which can have both inhibitory and stimulatory effects on immune cells. Disrupted immune responses and a state of chronic inflammation contribute to ovarian dysfunction in patients with PCOS (Shabbir *et al.* 2023). Few studies to date have investigated the effects of excess androgens on inflammation in women with PCOS. However, women with PCOS have an increased risk of developing cardiovascular diseases, such as atherosclerosis, and circulating immune cells have been investigated in this context in PCOS patients. A study was designed to investigate both the serum concentration of advanced glycation end-products (AGE) and the expression levels of AGE-specific receptors (RAGE) in circulating monocytes in 29 young women with PCOS. This study revealed that women with PCOS had increased concentrations of factors associated with atherosclerosis; serum concentrations of AGE were increased in PCOS compared to healthy individuals, and a positive correlation was identified between AGE proteins and testosterone concentrations. RAGE expression was also found to be increased in monocytes of PCOS women compared to controls (Diamanti-Kandarakis *et al.* 2005). AGE binding to RAGE in monocytes leads to activation, differentiation and the release of pro-inflammatory cytokines and chemokines such as TNF, IL1B and CCL2 (Kierdorf & Fritz 2013). Although this evidence is indirect, it suggests that chronically elevated androgens in PCOS patients may promote an inflammatory phenotype in circulating monocytes. However, further investigation is required to understand the relationship between elevated androgens and monocytes/macrophage function in women’s health.

### Neutrophils

Neutrophils are the most abundant leucocytes in human blood and are important effector cells in the innate immune system. In the presence of infection, they rapidly respond by trapping and killing pathogens through mechanisms such as phagocytosis, degranulation and the release of extracellular traps (Rosales 2018). Immunohistochemistry analysis has revealed that all neutrophil lineages, from precursors to mature neutrophils, express the cytoplasmic form of the AR in humans (Mantalaris *et al.* 2001, Markman *et al.* 2020).

Various studies have shown that androgens have an important role in neutrophil differentiation and function (Chuang *et al.* 2009, Marin *et al.* 2010, Inamdar & Jayamma 2012, Scalerandi *et al.* 2018). For example, studies utilising GARKO or mice with a testicular feminisation mutation (Tfm) that have loss of AR function, have a 90% reduction in neutrophils isolated from the bone marrow (Chuang *et al.* 2009). In particular, the proportion of myelocytes, metamyelocytes and mature neutrophils was reduced in GARKO mice. *In vitro* experiments in which granulocyte–myeloid progenitors were isolated from GARKO mice and exposed to retroviruses to restore the expression of AR revealed that the defects in neutrophil maturation could be restored by re-expressing AR, consistent with an essential role for AR in neutrophil differentiation (Chuang *et al.* 2009). These findings were further supported by another study in which mice were treated with the anabolic-androgenic steroid stanozolol (testosterone analogue), which increased myelocytes, metamyelocytes and mature neutrophils (Inamdar & Jayamma 2012). These studies demonstrate the important role of AR-mediated signalling in neutrophil development and maturation.

AR signalling has also been found to play a role in the ability of neutrophils to mount an efficient immune response to infection. GARKO studies demonstrated a reduction in neutrophil number resulting in a severely diminished capacity to survive bacterial challenge (Chuang *et al.* 2009). *In vitro* studies using human neutrophils revealed that testosterone treatment leads to a significant decrease in superoxide production, alterations in calcium mobilisation and altered NO production (Marin *et al.* 2010). Notably, modulation of human neutrophil function by androgens may be dose dependent, since neutrophils treated with 10 nM of testosterone exhibited increased phagocytic capacity while neutrophils treated with 10 μM of testosterone exhibited decreased microbicidal activity (Marin *et al.* 2010). In a rat model of bacterial prostate inflammation, testosterone-treated rats experienced higher neutrophil recruitment which was associated with increased tissue damage (Scalerandi *et al.* 2018). Interestingly, neutrophils exposed to exogenous testosterone exhibited diminished bactericidal ability as well as reduced myeloperoxidase activity (Scalerandi *et al.* 2018). This altered neutrophil profile was characterised by increased expression of IL-10 and TGFB1, which resemble the ‘N2-like’ neutrophil phenotype that has previously been demonstrated in tumours (Scalerandi *et al.* 2018). Thus, androgens reduce the overall effector response of neutrophils in experimental settings.

### Impact of androgens on neutrophils in disease

In the clinical setting, the effects of androgens on neutrophils can be inferred from scenarios that alter androgen bioavailability. For example, women with hyperandrogenism due to PCOS exhibit neutrophilia, and treatment with an anti-androgen attenuated neutrophilia (Ibáñez *et al.* 2005). Men with prostate cancer who received anti-androgen treatment, nilutamide or flutamide, suffered from severe neutropenia, which was reversed upon cessation of treatment, and neutrophil counts returned to normal (McDonnell & Livingston 1994, Eaton & Blackmore 2001). These studies demonstrate the impact androgens can have on the abundance of circulating neutrophils, but also that these effects can be context-dependent. Altering neutrophil abundance using androgens may have utility in certain clinical scenarios, but further studies will be required to determine the potential kinetics of these effects.

## Impact of androgens on the adaptive immune system

### T lymphocytes

T lymphocytes, also known as T cells, are an important adaptive immune cell type that is derived from CLPs in the bone marrow and is split into subtypes based on maturity and function. Naive T cells have yet to encounter their corresponding antigen and therefore are inactive (Kumar *et al.* 2018), but differentiate into an effector T cell following activation. CD8^+^ T cells, also known as cytotoxic T cells, kill target cells expressing a specific antigen by releasing perforin and granzymes into the immunologic synapse resulting in apoptosis of the target cell (LaRosa & Orange 2008). CD4^+^ T cells, also known as helper T cells, suppress immune responses to maintain homeostasis and prevent tissue damage. Further differentiation of CD4^+^ T cells takes place in the presence of certain cytokines and these subpopulations exhibit specific functions (Luckheeram *et al.* 2012). As seen in Fig. 2, both murine and human T cells are reported to express both the classic cytoplasmic and membrane-bound forms of the AR.

The effects of androgens on T cell development and function have been studied both *in vivo* using rodent models and *in vitro* using cell lines as outlined in Table 2. Several studies have utilised models of castration in male mice to deplete androgens and investigate the downstream effects of this depletion. It has been shown that castration leads to thymic enlargement and a significant decrease in the proportion CD4^−^ CD8^+^ thymocytes, which was reversed by exogenous testosterone treatment (Olsen *et al.* 1991). Studies have also shown that treatment with testosterone or methyltestosterone led to a decrease in the percentage of CD4^+^ CD8^+^ thymocytes (Olsen *et al.* 1991, Dulos & Bacchus 2001). Production of IL-4, IL-5 and interferon gamma (IFNG) by murine splenic T cells was reduced by DHT after anti-CD3 activation (Araneo *et al.* 1991). The same study revealed that androgen supplementation using physiologically relevant doses of DHT and DHEA in aged (>60 weeks) murine splenic T cells restored the capacity of this cell type to produce IL-2, IL-4 and IFNG to the same extent as younger mice (Araneo *et al.* 1991).

**Table 2:** A summary of primary research studies investigating the effects of androgens on T lymphocytes *in vitro* and *in vivo*.

Species	Cell type	Source	Sex	Purpose	Experimental design	*In vitro/in vivo*	Key findings	Analysis technique	Reference
Mouse	T lymphocytes	Spleen	Male and female	Investigate the effects of DHT on murine T cell production of lymphokines	Direct exposure of murine T cells to physiological relevant levels of DHT	*In vitro*	DHT administration reduced production of IL-4, IL-5 and IFNG after anti-CD3 activation	Lymphokine bioassays	Araneo *et al.* (1991)
Mouse	T lymphocytes	Thymus	Male	Investigate the functional immunological consequences of thymic regeneration after castration and whether or not this can be reversed via administration of testosterone	Male mice (8–10 weeks old) were castrated and then culled ~13 days post castration or were castrated and subcutaneously received 1 mg doses of testosterone cypionate every other day for three or four doses Thymocytes were isolated from both mouse models.	*In vivo*	Castration led to an enlarged thymus and a significant decrease in the proportion of CD8+ thymocytes. While testosterone replacement after castration resulted in thymic regression, a shift to expression of mature thymocyte phenotypes and a relative predominance of CD8+ T cells over CD4+ T cells	Flow cytometry	Olsen *et al.* (1991)
Mouse	Thymocytes	Thymus	Male and female	Investigate the mechanism behind androgen-induced thymic involution	Thymus organ cultures were produced from female mice and male mice that had been castrated at 3 weeks. Organ cultures were then treated with DHT and were used to prepare cell suspensions or were embedded in paraffin	*In vitro*	Administration of DHT to thymus organ cultures resulted in increased thymocyte apoptosis	Measurement of DNA fragmentation by ELISA and visualisation of apoptotic nuclei via ApopTag	Olsen *et al.* (1998)
Mouse	Thymocytes	Thymus	Female	Investigate the impact of androgen methyltestosterone on thymocytes	Mice were treated for 7 days with subcutaneous injections of methyltestosterone before the thymus was removed and thymocytes isolated	*In vivo*	Methyltestosterone administration significantly decreased the percentage of CD4+ CD8+ thymocytes	Flow cytometry	Dulos & Bacchus (2001)
Mouse	CD4+ T lymphocytes	Spleen	Male	Investigate the effect of androgens on murine T lymphocytes	Male mice were castrated and CD4+ T lymphocytes were isolated from the spleen (androgen deprivation) CD4+ T lymphocytes were cultured with IL-12 and 2 ng/mL of the androgen analogue R1881 (androgen supplementation)	*In vivo* and* in vitro*	Androgen deprivation *in vivo* led to significant gene expression changes in pathways involved in IFN signalling and Th1 differentiation. Further interrogation of the mechanisms in which androgens Th1 differentiation revealed that testosterone inhibits IL-12-induced Stat4 phosphorylation	Affymetrix microarray and western blot	Kissick *et al.* (2014)
Rat	CD4+ T lymphocytes	Spleen	Male	Investigate the impact of androgens on the differentiation and function of regulatory T cells	Leydig cells were co-cultured with splenic CD4+ T lymphocytes in the presence of a cell stimulation cocktail	*In vitro*	CD4+ T lymphocytes treated with androgens expressed Foxp3 and secreted IL-10 in a dose-dependent manner. This effect was abolished by the addition of flutamide (an anti-androgen)	Flow cytometry and ELISA	Fijak *et al.* (2015)

CD3, cluster of differentiation 3; CD4, cluster of differentiation 4; CD8, cluster of differentiation 8; DHT, dihydrotestosterone; ELISA, enzyme-linked immunosorbent assay; Foxp3, forkhead box protein 3; IFNG, interferon gamma; IL-4, interleukin 4; IL-5, interleukin 5; IL-10, interleukin 10; IL-12, interleukin 12; Th1, type 1 T helper cell.

Kissick *et al.* utilised various androgen modulation methods, including castration and administration of the androgen analogue R1881, to investigate the impact of androgens on murine CD4^+^ splenic T cells. These studies demonstrated that androgen depletion led to significant gene expression changes in pathways involved in IFNG signalling and T-helper cell differentiation. It was also demonstrated that androgen treatment significantly reduced IFNG production (Kissick *et al.* 2014). CD4^+^ T cells treated with androgens expressed FOXP3 and produced IL-10 in a dose-dependent manner, and this effect could be abolished by the addition of the anti-androgen flutamide (Fijak *et al.* 2015).

In summary, androgens largely reduce T cell number and dampen T cell responses through the reduced production of inflammatory mediators, such as IL-5 and IFNG, and the increased production of IL-10 (Araneo *et al.* 1991, Olsen *et al.* 1991, Olsen *et al.* 1998, Dulos & Bacchus 2001, Kissick *et al.* 2014, Fijak *et al.* 2015).

### Impact of androgens on T cells in disease

#### Autoimmunity

It is well known that males have reduced risk of developing various autoimmune diseases, such as multiple sclerosis (MS), systemic lupus erythematosus and rheumatoid arthritis (RA), when compared to females (Angum *et al.* 2020). To prevent the development of autoimmune diseases, the immune system must be able to recognise self-produced antigens without eliciting an immune response. This is known as self-tolerance, and loss of tolerance is known to underpin several autoimmune disorders (Zhang & Lu 2018). In the thymus, T-cell self-tolerance is enforced by the expression of the autoimmune regulator (Aire) gene, which promotes the expression of tissue-specific antigens (TSAs) by thymic epithelial cells. Immature thymocytes that come into contact with and recognise these TSAs with high affinity will be selectively removed from the population, preventing the maturation of self-reactive T cells. One study investigating sexual dimorphism in autoimmune disorders utilised mouse models and demonstrated that DHT treatment may have a protective effect against autoimmunity due to AR-dependent upregulation of *Aire* mRNA expression in thymic stromal cells. This increase in *Aire* mRNA expression leads to an upregulation in TSA expression and therefore negative selection of self-reactive T cells, limiting their release into the periphery (Zhu *et al.* 2016). The results of this study suggest that androgen therapy could be utilised to enhance *Aire* mRNA expression and therefore protect against autoimmunity, and opens new therapeutic avenues for autoimmune disorders.

#### Multiple sclerosis and experimental autoimmune encephalomyelitis

MS is an autoimmune condition that affects the brain and spinal cord as the immune system attacks the myelin that covers and protects nerve fibres in the central nervous system (CNS). MS is more predominant in women with a ratio of approximately 3:1 (Voskuhl *et al.* 2020). Experimental autoimmune encephalomyelitis (EAE) is a widely used mouse model of MS. EAE is a helper T cell-mediated autoimmune disease that causes inflammation in the CNS characterised by infiltration of monocytes and T cells, demyelination and axonal loss (Robinson *et al.* 2014).

Insertion of a DHT pellet in female mice resulted in significantly less severe EAE when compared to mice that received a placebo pellet. Furthermore, T cells specific for myelin basic protein from females treated with DHT expressed significantly higher levels of IL-10 than controls (Dalal *et al.* 1997). Similar results were found in another study where female mice were treated with physiological doses of DHT (5 mg), which increased IL-10 production in AR^+^ CD4^+^ splenic T cells (Liva & Voskuhl 2001). Furthermore, administration of DHT 45 days after the induction of EAE in male rats led to an improvement in clinical scores and decreased inflammation in the spinal cord (Giatti *et al.* 2015). Taken together, these studies indicate that androgens have a protective effect in a mouse model of EAE that is in part mediated by increased IL-10 production by autoantigen-specific CD4^+^ T cells.

#### Prostate cancer and benign prostatic hyperplasia

Androgen deprivation therapy (ADT) is the main treatment for patients with prostate cancer; however, prostate cancer often recurs and becomes hormone therapy resistant. Recent evidence suggests that shortly after androgen deprivation, there is an expansion of T cells in both mouse models and prostate cancer patients. Wang *et al.* set out to investigate the role that different T cell subtypes play in hormone therapy-resistant prostate cancer. This study demonstrated that there was a novel population of CD4^low^HLA-G^+^ T cells that expanded in prostate cancer patients post ADT. These cells were found to promote the growth of prostate cancer cells through androgen-independent mechanisms that modulate the migration and activity of CD11b^low^F4/80^hi^ macrophages. It was found that after androgen deprivation, there was an elevation of PGE2-EP2 signalling which led to the polarisation of this novel population of CD4^low^HLA-G^+^ T cells. Inactivation of PGE2, via celecoxib, during the appearance of CD4^low^HLA-G^+^ T cells significantly suppressed the onset of hormone therapy-resistant prostate cancer, suggesting that combining ADT with PGE2 inhibition may be effective in prostate cancer (Wang *et al.* 2018).

ADT has also been shown to sensitise prostate cancer patients to effective checkpoint blockade due to the enhanced function of CD8+ T cells. Inhibition of AR signalling via ADT prevented the exhaustion of CD8+ T cells and increased IFNG expression, resulting in improved responsiveness to programmed cell death protein 1 (PD-1) targeted treatment. The AR has the capacity to bind *Ifng* directly, repressing its expression, and preventing this binding using enzalutamide significantly increased IFNG production in CD8+ T cells. These findings demonstrate a novel mechanism of immunotherapy resistance in prostate cancer (Guan *et al.* 2022). This knowledge can be expanded to incorporate different tumour and treatment types to investigate this mechanism in other areas of cancer research.

Another condition that is androgen sensitive is benign prostate hyperplasia (BPH), which is the non-malignant growth or hyperplasia of prostate tissue (Roehrborn 2005). Emerging evidence from clinical studies suggests that androgens may be linked to the infiltration of T cells in BPH, but T cell subset information and potential mechanisms of this action are unknown. To investigate if DHT increases the infiltration of T cells via BPH epithelial cells, prostate tissues were collected from 64 BPH patients after transurethral resection of the prostate, split into two groups: (1) no medication history and (2) treated with finasteride (5α-reductase inhibitor) 5 mg daily for at least 6 months prior to surgery. Tissues from BPH patients treated with finasteride had significantly higher infiltration of CD8^+^ T cells into the prostate with no changes in CD4^+^ T cells and increased epithelial CCL5 expression (Fan *et al.* 2014). This study illustrated that intraprostatic DHT may have an important role in the regulation of immune responses through the recruitment of CD8^+^ T cells by human prostatic epithelial cells via their secretion of CCL5 (Fan *et al.* 2014). Thus, androgen availability may affect T cell composition and function in the prostate and impact disease outcomes in BPH and hormone therapy-resistant prostate cancer.

### B lymphocytes

B lymphocytes, also known as B cells, are adaptive immune cells that provide humoral immunity through antibody production. B cells develop from CLPs in the bone marrow and undergo a series of differentiation steps that result in the maturation and formation of the B cell receptor (BCR) (Cano & Lopera 2013). B cells can express a range of immunoglobulin (Ig) receptors including IgM, IgG, IgA, IgE and IgD, and these antibody molecules exert different immune functions such as neutralisation, opsonisation and high-affinity receptor-mediated killing of pathogens (Alberts *et al.* 2002). B cell progenitors, but not mature or peripheral B cells express, the cytoplasmic form on AR. B cells are therefore sensitive to the actions of androgens primarily during their development in the bone marrow (Benten *et al.* 2002*a*).

Androgens play an important role in B cell maturation and homeostasis. Altuwaijri *et al.* investigated the effect of androgens on B cell function using a variety of male mouse models. These models included castration, GARKO *(Actb^Cre^Ar^flox^*) mice, B-cell-specific ARKO (BARKO – *Cd19^Cre^Ar^flox^*) mice, and Tfm mice to demonstrate that AR deficiency led to higher B cell numbers in the blood and the bone marrow due to increased proliferation of B cell precursors in the bone marrow. It was also seen that B cells with deficient AR had higher resistance to apoptosis and that implantation of a DHT pellet restored B cell numbers in castrated mice, but not GARKO mice, supporting the hypothesis that B cell maturation is androgen mediated and AR dependent (Altuwaijri *et al.* 2009). *In vitro* studies investigating the effect of androgens on B cell function demonstrated that testosterone treatment inhibited the production of IgG and IgM by B cells. This reduced production could be partially restored by the addition of exogenous IL-6. This study demonstrated that androgens could alter B cell function through the inhibition of antibody production (Kanda *et al.* 1996).

Ellis *et al.* investigated the effect of castration-dependent androgen ablation on B cell lymphopoiesis and the phenotype of peripheral B cell populations. Castration led to a significant increase in spleen weight and the total number of peripheral blood B cells (Ellis *et al.* 2001). Further experiments were carried out to determine whether the increase in peripheral blood B cells was due to an increase in the number of mature lymphocytes and/or due to an increase in newly formed B cells that have emigrated from the bone marrow. Analysis of peripheral blood B cells from castrated mice revealed an increase in mature B cells as well as B cells that had low expression of B220 and high expression of CD24. These markers identify newly emigrated immature B cells which proportionally were increased to a greater extent than mature B cells. This demonstrates that the observed increase in circulating B cells after castration is in part due to increased bone marrow egress of immature cells which remained elevated up to 54 days after castration (Ellis *et al.* 2001). These results demonstrate that loss of androgens can increase B cell lymphopoiesis and emigration of immature B cells from the bone marrow; however, it is unclear if this is a direct or indirect effect. An increase in the number of splenic B cells was demonstrated in a study that utilised general ARKO male mice (ubiquitous Pgk1; *Pgk1^Cre^Ar^flox^*). These mice also had increased serum levels of BAFF, a cytokine that is an essential splenic B cell survival factor (Wilhelmson *et al.* 2018). Increased serum BAFF levels are detected in healthy men with low testosterone, which suggests an association between testosterone, BAFF production, and B cell number. Taken together, these studies demonstrate the importance of androgens and AR signalling in the maturation and homeostasis of B cells.

### Impact of androgens on B cells in disease

#### Rheumatoid arthritis

RA is a chronic inflammatory autoimmune disorder that primarily affects the joints. RA is caused by abnormalities in the cellular and humoral immune response resulting in the generation of autoantibodies against targets, such as rheumatoid factors (RF). RA also results in the migration of T and B cells into the synovium and activation of the innate immune system within the affected tissues (Scherer *et al.* 2020). Male RA patients are reported to have low androgen levels (Cutolo *et al.* 1984, Spector *et al.* 1988), and prostate cancer patients receiving ADT have an altered risk of developing RA. This is characterised by a higher 5-year incidence rate of RA diagnosis in patients who received ADT compared to those who did not (Yang *et al.* 2018). To investigate the role of B cell AR in the development of autoimmune diseases, B cell ARKO mice (*Cd19^Cre^Ar^flox^*) were generated, and serum antibody levels were analysed. B cell ARKO mice had increased levels of IgG3 in their serum compared to wildtype mice as well as significantly elevated levels of autoantibodies that bind DNA (double-stranded DNA-IgG), which is a hallmark of autoimmune susceptibility. GARKO and B cell ARKO mice also show increased susceptibility to collagen-induced arthritis (CIA) with increased levels of basal IgG-RF autoantibodies and significant CIA manifestations, including enlarged synovial membrane and lymphocyte infiltration, 10 weeks post-bovine collagen II immunisation (Altuwaijri *et al.* 2009). Taken together, the data from clinical studies as well as the results generated in these B cell-deficient mouse models suggest that AR may be a plausible target for new therapies to treat autoimmune diseases, such as RA.

## Final summary

**Figure 3:**
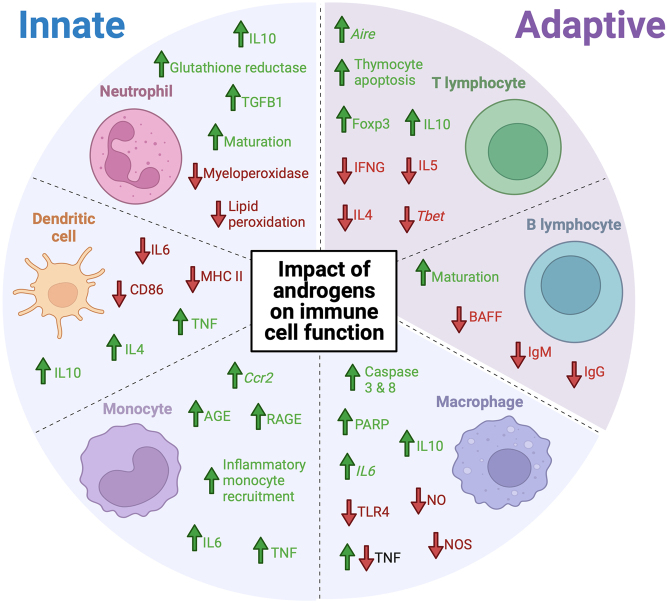
The impact of androgens on immune cell function in health and disease. In general, androgens tend to exhibit an anti-inflammatory effect on immune cells. This is demonstrated by the upregulation of inflammatory mediators, such as IL-10, in various immune cell types including T lymphocytes (Fijak *et al.* 2015), macrophages (D’Agostino *et al.* 1999), dendritic cells (Thompson *et al.* 2017) and neutrophils (Scalerandi *et al.* 2018) in the presence of androgens. It has also been demonstrated that androgens can reduce production of several inflammatory mediators, such as IFNG, IL-4 and IL-5 in T lymphocytes (Araneo *et al.* 1991) and NO in macrophages (D’Agostino *et al.* 1999, Friedl *et al.* 2000). Androgen have also been found to promote immune cell maturation and differentiation in cells such as T lymphocytes (Olsen *et al.* 1991), B lymphocytes (Altuwaijri *et al.* 2009) and neutrophils (Chuang *et al.* 2009, Inamdar & Jayamma 2012). Green arrows represent upregulation or increase. Red arrows represent downregulation or decrease. For abbreviations see text. Created with BioRender.com.

Androgens and AR play an important role in the modulation of immune responses. Several innate and adaptive immune cell subsets express the cytoplasmic and/or membrane-bound form of AR and therefore have the capacity to be modulated by androgens (Fig. 2). Androgens/AR can modulate the development of immune cell subsets in both primary (bone marrow) and secondary (spleen) lymphoid organs with important roles in neutrophil and B cell maturation. Androgens exert their effect on immune function through various mechanisms in a cell-specific manner (Fig. 3). This includes a reduction in immune cell numbers, such as T cells, polarisation of monocyte and macrophage towards an anti-inflammatory phenotype, or inhibiting immune cell activation in the case of DCs. Androgens are therefore broadly associated with having immunosuppressive effects.

Changing the bioavailability of androgens in both health and disease can therefore alter immune cell function and responses. This has been explored in the context of sexual dimorphism, where the simple dichotomy of males as a high androgen state and females as a (relatively) low androgen state has been used to account for androgens having a role in the presentation/progression of sexually dimorphic diseases. However, variation in androgen bioavailability is not solely accounted for by sex. Endocrinopathies in both males and females can result in elevated (e.g. PCOS, congenital adrenal hyperplasia) or lowered (e.g. hypogonadism, premature ovarian insufficiency) androgens. Furthermore, surgical or medical castration can ablate endogenous androgens, and androgen replacement therapies are utilised in prostate cancer treatment and as part of post-menopausal hormone replacement therapy. Thus, understanding how androgens directly and indirectly affect immune cell function is key to determining their potential role in immune disorders.

Studies investigating the effects of androgens on immune cells in disease have shown that they are context-dependent and can differ even in the same immune cell type depending on the disease state. For example, it has been shown that androgens have an inhibitory effect in cutaneous wound healing by supressing pro-repair macrophages and production of TNF, whereas, in the context of asthma, it has been demonstrated that DHT treatment in experimental models reduces inflammation and promotes polarisation of alveolar macrophages into a pro-repair phenotype. These studies demonstrate that the impact of androgens on immune cells is highly nuanced, which can impact both the efficacy and specificity of androgen-targeting treatments, whether as direct therapies or as an indirect consequence of altered endocrine states.

## Future directions

Androgen therapy impacts immune cells but has largely not been used to target immune function directly. Beyond endogenous ligands and classic antagonists, selective androgen receptor modulators (SARMs) have been developed, which have both agonist and antagonist effects on AR. SARMs exploit context-dependent impacts of AR signalling and have been developed to facilitate tissue-specific benefits without off-target side effects. There is currently no SARM approved by the Food and Drug Administration; however, there are many ongoing phase I and II clinical trials examining these treatment strategies for a range of conditions, including breast cancer, prostate cancer, BPH, and chronic obstructive pulmonary disease. This is a promising treatment avenue that has the potential to treat a wide spectrum of disorders, including those listed above, and others such as osteoporosis, muscle wasting and hypogonadism (Christiansen *et al.* 2020). Detailed information regarding both approved and ongoing clinical trials utilising androgen modulation as a treatment strategy is discussed in detail elsewhere (Gamat & McNeel 2017, Ben-Batalla *et al.* 2020, Christiansen *et al.* 2020). Importantly, none have a primary indication for modulating immune cell-mediated diseases.

One challenge of investigating the effects of androgens in androgen-responsive tissues, such as the prostate, is that while androgens might mediate specific effects on immune cells, there are other cell types in the tissue, such as epithelial and stromal cells, that will also express the AR. Thus, delineating the effects of androgens/AR activation in tissue homeostasis and disease contexts will need to take this into account to understand how the cells in the tissue are being directly and indirectly modulated by androgens. Androgens may induce an anti-inflammatory phenotype in one cell type, but if AR activation in another cell type causes the release of mediators that bring about the opposite effect, the net result may not necessarily be anti-inflammatory. Further studies utilising *in vitro* techniques and conditional knockout models *in vivo* would be required to explore the direct regulation of androgens on cell types of interest and to identify downstream mediators of androgen signalling. Future research should take into account the more complete context of hormone availability, receptor expression and sites of cell/tissue action when exploring roles for androgens in modulating immune cell function.

## Conclusion

Androgens can have a wide variety of effects on immune cells, and these can differ depending on disease context and anatomical location. There remain various unanswered questions surrounding the effect of androgens on the immune system, including effective ways to modulate androgen-regulated processes and understanding androgen balance in the context of endocrinopathies and disease states. These will need to be accounted for to progress drug development and clinical use of treatments that target androgen action in inflammatory contexts. Embracing androgens as immunomodulatory agents will help to improve understanding of endocrine aspects of inflammatory disorders and open new avenues for repurposing and developing androgen therapies to modulate immune cell function.

## Declaration of interest

The authors declare that there is no conflict of interest that could be perceived as prejudicing the impartiality of this review.

## Funding

This work was supported by the Wellcome Trusthttp://dx.doi.org/10.13039/100010269 (Fellowship 220656/Z/20/Z to DAG), the Medical Research Councilhttp://dx.doi.org/10.13039/501100000265 studentship (MR/W006804/1) and Medical Research Councilhttp://dx.doi.org/10.13039/501100000265 grant (MRC/IAA/002).
